# The Effect of Metronidazole versus a Synbiotic on Clinical Course and Core Intestinal Microbiota in Dogs with Acute Diarrhea

**DOI:** 10.3390/vetsci11050197

**Published:** 2024-04-29

**Authors:** Helene Stübing, Jan S. Suchodolski, Andrea Reisinger, Melanie Werner, Katrin Hartmann, Stefan Unterer, Kathrin Busch

**Affiliations:** 1 Small Animal Clinic, Centre for Clinical Veterinary Medicine, Ludwig-Maximilian University Munich, 80539 Munich, Germanyhartmann@lmu.de (K.H.); kathy.busch@gmx.de (K.B.); 2 Gastrointestinal Laboratory, School of Veterinary Medicine and Biomedical Sciences, Texas A&M University, College Station, TX 77840, USA; jsuchodolski@cvm.tamu.edu; 3 Clinic for Small Animal Internal Medicine, Vetsuisse Faculty, University of Zurich, 8057 Zurich, Switzerlandstefan.unterer@uzh.ch (S.U.)

**Keywords:** antibiotic, *C. perfringens*, *C. hiranonis*, *E. coli*, *E. faecium*, probiotic, intestinal microbiome, clinical improvement, canine

## Abstract

**Simple Summary:**

The usefulness of metronidazole for acute diarrhea treatment in dogs is discussed. The role of *Clostridium perfringens* and *Escherichia coli* as enteropathogens in acute uncomplicated diarrhea (AD) in dogs is controversial, whereas some beneficial bacteria, such as *Clostridium hiranonis*, are important members of the normal intestinal microbiota. In this study, the effects of metronidazole and a synbiotic on the clinical course and core intestinal microbiota in dogs with acute diarrhea were compared. No significant benefit of metronidazole was observed regarding the clinical course. Metronidazole had no effect on *C. perfringens* concentration but resulted in significant increased concentration of *E. coli*, an increased Dysbiosis Index, and a reduction in *C. hiranonis* concentration. In conclusion, in contrast to synbiotic treatment, metronidazole treatment negatively impacts the microbiome without affecting clinical outcomes.

**Abstract:**

The usefulness of antibiotics in dogs with acute diarrhea (AD) is controversial. It is also unclear what effect metronidazole has on potential enteropathogens such as *Clostridium perfringens* and *Escherichia coli*. Thus, the aim of this study was to evaluate the effect of metronidazole vs. a synbiotic on the clinical course and core intestinal bacteria of dogs with AD. Twenty-seven dogs with AD were enrolled in this prospective, randomized, blinded clinical trial and treated with either metronidazole (METg) or a synbiotic (SYNg; *E. faecium* DSM 10663; NCIMB 10415/4b170). The Canine Acute Diarrhea Severity (CADS) index was recorded daily for eleven days. Bacteria were quantified using qPCR. Data were analyzed using mixed models with repeated measures. A higher concentration of *E. coli* was observed in the METg group vs. the SYNg group on Day 6 (*p* < 0.0001) and Day 30 (*p* = 0.01). Metronidazole had no effect on *C. perfringens. C. hiranonis* was significantly lower in the METg group than in the SYNg group on Days 6 and 30 (*p* < 0.0001; *p* = 0.0015). No significant differences were observed in CADS index, fecal consistency, or defecation frequency between treatment groups (except for the CADS index on one single day). In conclusion, metronidazole negatively impacts the microbiome without affecting clinical outcomes. Thus, synbiotics might be a preferred treatment option for dogs with AD.

## 1. Introduction

Acute uncomplicated diarrhea in dogs (AD) is a common reason for veterinary consultations [1,2]. AD can be differentiated from acute hemorrhagic diarrhea syndrome (AHDS), as dehydration and bacterial translocation can lead to a complicated course [3]. Typically, most cases of AD are mild and dogs can usually be treated as outpatients, requiring no specific treatment [4,5,6,7]. AD occurs suddenly and persists for fewer than 7 days.

The causes of AD are usually unknown. Dietary indiscretion, inappropriate diet, abrupt dietary changes, dietary intolerance and hypersensitivities, stress, and medications irritating the gastrointestinal tract (e.g., non-steroidal anti-inflammatory drugs) are among the non-infectious risk factors [8,9,10]. Transient self-limiting gastrointestinal infections are another potential cause of AD in dogs. In this context, *Clostridium perfringens* (*C. perfringens*) and *Escherichia coli* (*E. coli*) are frequently mentioned as potential enteropathogens. However, bacterial infections are generally rare in dogs and previous studies have shown that *C. perfringens* and *E. coli* can also be detected in healthy dogs [11,12]. Nevertheless, the identification of these bacteria in fecal samples of dogs with AD is often used as an indication for antibiotic treatment [10], which is initiated in 49 to 71% of dogs presenting with AD [13,14,15].

The most frequently prescribed antimicrobial agent in dogs with AD is metronidazole, which comprised 47% of all antimicrobial prescriptions in a study from the United Kingdom [13]. Metronidazole is a 5-nitroimidazole drug and is used in the management of a variety of infectious diseases, especially for infections with anaerobic bacteria and protozoa [3,16,17]. Metronidazole is a concentration-dependent bactericidal drug and is widely distributed throughout the body [3]. The activation of this prodrug includes a reduction of the nitro group within the target cell. The reductases of anaerobic or microaerophilic microorganisms explains the selectivity of metronidazole. The interaction of generated metabolites with the DNA leads to cell death of bacteria and protozoa [17].

On the World Small Animal Veterinary Association’s (WSAVA) list of essential medicines, metronidazole is listed as an antibiotic that satisfies primary health care and welfare of cats and dogs and is recommended for management of selected bacterial and protozoal enteric infections [18]. However, there is a routine prescription of metronidazole in AD. The efficacy of metronidazole in the treatment of AD is highly controversial, especially considering the development of resistant bacterial strains and potential negative effects on the intestinal microbiome (e.g., severe dysbiosis due to its anaerobic spectrum) [19,20,21,22]. In detail, a significant reduction in *Clostridium hiranonis* (*C. hiranonis*) concentration after the administration of metronidazole has already been demonstrated [19]. C. *hiranonis* has been reported to be beneficial in dogs due to its conversion of primary to secondary bile acids, which is important in the regulation of *C. difficile* and *C. perfringens* in both dogs and humans [23,24].

Synbiotics have probiotic and prebiotic properties. The World Health Organization (WHO) defined them as live strains of strictly selected microorganisms that, when administered in adequate amounts, confer a healthy benefit on the host. Prebiotics are nondigestible food ingredients that beneficially affect the host by selectively stimulating growth and/or activity of one or a limited number of bacteria to improve host health [25,26]. The purpose of synbiotics is to overcome some possible difficulties in the survival of probiotics in the gastrointestinal tract. A combination of both components in a single product should ensure a superior effect compared to the activity of the probiotic or prebiotic alone [27,28]. The purpose of synbiotic treatment in AD treatment is to support self-limiting characteristics via positive modulation of the intestinal microbiome; therefore, synbiotics are useful alternatives to antibiotic treatment. However, the results of different studies comparing metronidazole to probiotics/synbiotics and a placebo are inconsistent. One clinical trial suggested that metronidazole treatment, compared to a placebo, can shorten the duration of AD in dogs (mean 2.1 versus 3.6 days) [6], while other clinical trials failed to show a faster clinical improvement in response to metronidazole compared to probiotic, placebo, or nutraceutical treatment [4,7].

Therefore, the aim of this study was to evaluate the impact of metronidazole compared to that of the administration of a synbiotic on the clinical course as well as the abundances of specific bacterial species, such as *C. perfringens*, *E. coli*, and *C. hiranonis*, and the intestinal microbiome in general in dogs with AD.

## 2. Materials and Methods

This study was designed as a prospective, blinded, randomized clinical trial and was approved by the ethical committee of the Centre for Clinical Veterinary Medicine Ludwig-Maximilians University, Munich (reference 205-06-03-2020). Dogs were recruited from two small animal clinics in Munich, Germany, between October 2020 and January 2023.

Twenty-seven dogs with acute diarrhea < 5 days in duration, a fecal consistency score of at least 2 on the Canine Acute Diarrhea Severity index (CADS index; Table 1, [29]), a bodyweight between 5 and 50 kg, and a minimum age of 9 months were enrolled.

Dogs with a history of chronic or recurrent gastrointestinal signs or treated with antibiotics or probiotics within 30 days or anti-inflammatory drugs within 7 days before presentation were excluded. Dogs that met any of the following criteria indicating a complicated form of acute gastrointestinal disease were also excluded: hemorrhagic diarrhea, signs of systemic inflammation or sepsis (Table 2), severe illness (e.g., depressed mental status, moderate to severe abdominal pain), or significant dehydration prompting hospitalization (Table 2).

Relevant information regarding the medical history, such as any past diarrheic episodes, other diseases or chronic illnesses, vaccination and deworming routine, exact description of the current AD episode, additional clinical signs (e.g., vomiting), previous drug administration, intake of foreign material or snow, presence of stress-related factors, and diet or dietary changes, was gathered in a standardized fashion.

The minimal database for diagnostic work included a fecal examination for endoparasites using fecal flotation and antigen testing for Giardia (SNAP Giardia Test, Idexx GmbH, Kornwestheim, Germany), and a complete blood cell count (ProCyte Dx, Idexx GmbH, Kornwestheim, Germany) was performed.

Dogs of both groups received the same standardized treatment, including maropitant (Prevomax, Dechra Veterinary Products Deutschland GmbH, Aulendorf, Germany) as an antiemetic (1 mg/kg given once subcutaneously) and metamizole (Novaminsulfon, Ratiopharm, Ulm, Germany) as an analgesic (30 mg/kg per os q8h for 2 days), and were fed the same high-fiber gastrointestinal diet (Gastrointestinal Biome dry, Hill’s Pet Nutrition GmbH, Hamburg, Germany) for 7 days. Owners were instructed to feed only the prescribed diet and not to feed treats and table scraps during study period.

Additionally, depending on their randomization (list created by an online research randomizer: www.graphpad.com/quickcalcs/randomize1.cfm, accessed on 1 September 2020), dogs of the metronidazole treatment group (METg) received metronidazole (Metrobactin, Dechra Veterinary Products Deutschland GmbH, Aulendorf, Germany) per os at 10 to 20 mg/kg BW q12h for 7 days, and dogs of the synbiotic treatment group (SYNg) received a synbiotic agent (NutraPro^®^K9, *E. faecium* DSM 10,663 NCIMB 10415/4b1707, dextrose, mannan-oligosaccharides, brewer’s yeast, inulin, minerals, and hydrolyzed poultry liver, NutraPet Systems Deutschland GmbH, Schlaitdorf, Germany) per os at 10^8^ CFU/kg BW q12h for 7 days. For blinding, identical capsules containing either metronidazole or the synbiotic were used, and every dog received the same number of capsules according to its predefined weight group.

At presentation, the attending veterinarian assessed the clinical disease activity by using information from the patient’s history and physical examination (CADS index day 0). During the following 10 days (day 1 to day 10), the owner evaluated the disease activity score at home. The CADS index includes 5 parameters (activity, appetite, vomiting times/day, fecal consistency, defecation frequency times/day) with 0 to 3 points for each parameter, and for a maximum possible value of 15 points. The severity levels of the CADS index were defined as nonsignificant (0–3), mild (4–5), moderate (6–8), or severe (≥9). To obtain more objective data on the parameter fecal consistency (FC) of the CADS index, the Purina Fecal Scoring Chart was used (PFS; https://freedomservicedogs.org/wp-content/uploads/2022/04/Purina-Fecal-Scoring-Chart.pdf, accessed on 1 September 2020), whereas PFS 2–3 = CADS index FC 0; PFS 4–5 = CADS index FC 1; PFS 6 = CADS index FC 2; and PFS 7 = CADS index FC 3 were defined. For dogs that defecated more than once per day, the FC score was calculated as the average of all defecations that day.

Natural, passed fecal samples were collected from each dog on day 0 before starting treatment and on day 6 and day 30. Aliquots for analysis were frozen at −80 °C within a few hours following collection (<6 h) and were subsequently sent as batches on dry ice to the Gastrointestinal Laboratory at Texas A&M University.

The Dysbiosis Index (DI) is a mathematical model used to quantify intestinal dysbiosis in fecal samples from dogs. To calculate the DI, an individual qPCR assay was performed for 7 bacterial taxa (*Faecalibacterium* spp., *Turicibacter* spp., *Streptococcus* spp., *E. coli*, *Blautia* spp., *Fusobacterium* spp., and *C. hiranonis*) and the total bacteria as described previously [30]. The DI is a single numeric value, and a reference interval from −9.1 to 9.3 had been previously established based on 116 healthy dogs. A DI < 0 was defined as no shift in the overall diversity. If individual bacterial groups were outside the reference interval, this was suggestive of minor changes. Values between 0 and 2 were defined as a mildly increased DI, suggesting a mild to moderate shift in the overall diversity. A DI ≥ 2 was defined as significantly increased, consistent with a major shift in overall diversity [5]. This classification has been shown to accurately predict microbiome shifts, as assessed by DNA shotgun sequencing [31]. Another study demonstrated that when the abundance of *C. hiranonis* (in log10) is >4.5, essentially all primary bile acids are converted to secondary bile acids [32].

The abundances of the *C. perfringens* 16S rRNA gene, *C. perfringens* enterotoxin gene, and *C. perfringens NetF* gene in feces were analyzed via qPCR assays as previously described [5,11]. The reference intervals for healthy dogs had been established previously based on data from 120 healthy dogs. The reference interval of *C. perfringens* was defined from 1.1 to 6.5, and of *C. perfringens* enterotoxin from 1.8 to 5.1. *C. perfringens* toxin *NetF* and *Clostridium difficile* were considered negative. The qPCR results were expressed as the log amount of DNA (fg) for each bacterial group per 10 ng of isolated total DNA.

Power analysis indicated that at least 8 dogs per group needed to be included in the study to detect a difference of 2 points in the CADS index between treatments (estimated SD of 1.5, power of 80% and *p* < 0.05). Statistical analyses were performed using GraphPad Prism (GraphPad Prism c9.0, GraphPad Software, San Diego, CA, USA). Data were evaluated for normality by the Anderson-Darling test.

Differences between the groups in terms of sex, age, bodyweight, and breed were evaluated with the Mann-Whitney U test. The course of the CADS index and those of individual variables, the DI, and individual bacteria were analyzed by mixed effect analysis with multiple comparisons for comparison between the METg and the SYNg.

(The significance level chosen for determining statistical significance was *p* < 0.05).

## 3. Results

### 3.1. Study Population

A total of 27 dogs were included in the study (METg, *n* = 15; SYNg, *n* = 12). There was no significant difference between the groups at baseline in age, sex, bodyweight, or breed distribution (Table 3 and Table 4).

Dogs presented with a median duration of diarrhea of 1 day (METg range 1–5 days; SYNg range 1–4.5 days). Thirteen (48%) dogs experienced vomiting in addition to diarrhea. Nineteen (73%) of the owners reported that their dog might have ingested an inadequate substance or that they had observed an intake. A stressful experience in the last few days before presentation leading up to AD was reported by 5 owners (19%). Twenty-six dogs were fed a commercial diet, and one dog was fed a commercial vegan diet. Treats and table scraps were given to 17 dogs (63%). Eleven (41%) dogs were dewormed regularly, with the last deworming occurring a median of 3.2 months ago (range 1–24 months). Sixteen (59%) dogs were irregularly dewormed. No dog suffered from another acute disease in the last 30 days. In 10 (37%) dogs, a chronic nongastrointestinal condition was documented. Medication in the last 30 days was given to 8 (27%) dogs, and 3 (11%) dogs were receiving long-term medication. Nineteen (70%) owners reported several previous episodes of diarrhea, and the median gap to the last episode of AD was 4.2 months (range 2–8).

### 3.2. Treatment Efficacy

None of the dogs in either group required an additional medical treatment or a dietary adjustment during the study time or follow-up period.

**Figure 1:**
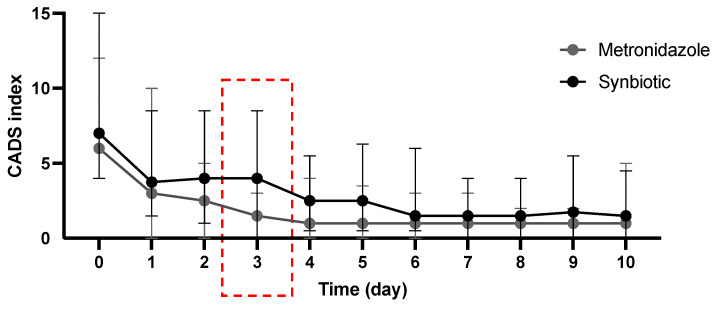
Clinical evaluation of clinical signs according to the Canine Acute Diarrhea Severity (CADS) index. The indices included the variables activity, appetite, vomiting (times/day), fecal consistency, and frequency of defecation (times/day). Each variable is scored from 0 to 3, and the sum of the scores yields a total cumulative score. Dots show the median, and error bars show the range. No statistically significant difference was observed between the METg and SYNg on any of the days except day 3 (red box; *p* = 0.02).

**Figure 2:**
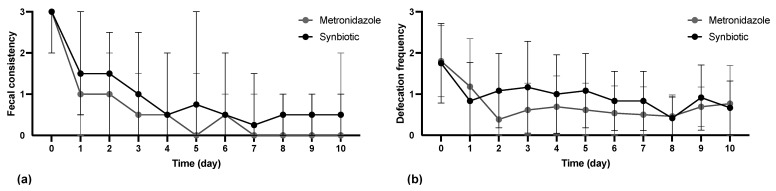
(**a**) Clinical evaluation of the parameter fecal consistency (FC) of the CADS index using the Purina Fecal Scoring Chart (PFS). The CADS index (FC) was scored from 0 to 3, with PFS 2–3 = CADS index FC 0; PFS 4–5 = CADS index FC 1; PFS 6 = CADS index FC 2; and PFS 7 = CADS index FC 3. Dots show the median, and error bars show the range. No significant difference in the CADS index FC was observed between the metronidazole and synbiotic treatment groups. (**b**) Clinical evaluation of the parameter defecation frequency (DF) according to the CADS index. The scores ranged from 0 to 3, with 0 = 1; 1 = 2–3; 2 = 4–5; and 3 = >5 times/day. The dots show the means, and the error bars show the SDs. No significant difference in the CADS index DF was observed between the metronidazole and synbiotic treatment groups.

The median CADS index on day 0 was 6.5 (range 4–15). No statistically significant difference in the CADS index was observed between the METg and the SYNg on any of the days except day 3 (*p* = 0.02; Figure 1). On day 3, more dogs in the SYNg had reduced appetite and activity. There was no significant difference on any day during the study period between the METg and the SYNg regarding diarrhea-specific parameters FC (Figure 2a) and defecation frequency (DF) (Figure 2b).

### 3.3. Microbiome Analysis

**Figure 3:**
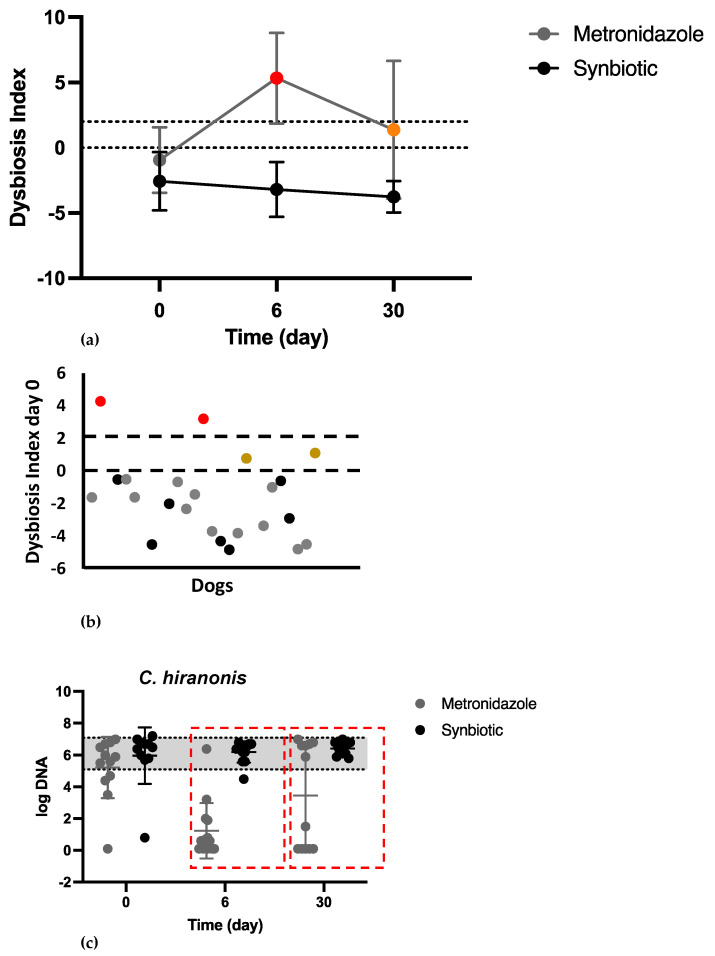
(**a**) The DI over time. The dots show the means, and the error bars show the SDs (**a**,**c**). A DI < 0 indicates a normal microbiome ((**b**) black dots) or minor changes ((**b**) gray dots; when at least one bacterial taxon is outside the reference interval), a DI of 0 to 2 indicates mild to moderate changes ((**a**,**b**) brown dots), whereas a DI ≥ 2 indicates significant changes ((**a**,**b**) red dots). There was a significant difference in the DI on day 6 (*p* < 0.0001) and day 30 (*p* = 0.0004) between the two groups. (**b**) The DI on day 0. Most of the dogs had a normal DI or minor changes (gray dots) on the day of presentation. (**c**) Concentration of *C. hiranonis* over time. The *y*-axis represents the log DNA/g feces; the gray area shows the reference interval of the individual bacterial strains. Although there was no difference on day 0, a significant difference in *C. hiranonis* abundance was observed on day 6 and day 30 (red boxes; *p* < 0.0001; *p* = 0.0015).

There was no significant difference in the fecal DI on day 0 between the two groups. Most of the dogs had a normal DI (DI < 0) or minor changes (DI < 0) on the day of presentation (Figure 3b). There was a significant difference in the DI between the two groups on day 6 (METg mean of 5.3 (2.63); SYNg mean of −3.1 (−2.02); *p* < 0.0001) and day 30 (METg mean of 1.3 (4.69); SYNg mean of −3.7 (−1.15); *p* = 0.0004; Figure 3a). On the last recorded day, 33% of the dogs in the METg still had an increased DI, while all dogs in the SYNg had a DI < 0, with 58% of these dogs showing minor changes on day 30.

No significant difference in *C. hiranonis* abundance was observed on day 0 between the two groups. On day 0, 78% of all dogs had *C. hiranonis* within the reference range. There was a significant difference in *C. hiranonis* abundance between the metronidazole treatment group and the synbiotic treatment group on day 6 and day 30 (day 6, METg mean 1.6 log DNA/g feces (SD 1.68); SYNg mean 6.2 log DNA/g feces (SD 0.64), *p* < 0.0001; day 30, METg mean 3.4 log DNA/g feces (SD 3.05); SYNg mean 6.4 log DNA/g feces (SD 0.37), *p* = 0.0015). *C. hiranonis* was <4.5 log DNA/g feces on day 6 in 93% of the dogs and on day 30 in 42% of the dogs in the METg (Figure 3c).

Three bacterial taxa, quantified by PCR, *Turicibacter* spp., *Bifidobacterium* spp., and *Streptococcus* spp., were not significantly affected by metronidazole compared to synbiotic treatment, while a significant decrease in the abundances of *Faecalibacterium* spp. (*p* = 0.0015), *Fusobacterium* spp. (*p* = 0.0048), and *Blautia* spp. (*p* = 0.02) was observed due to metronidazole during the study.

**Figure 4:**
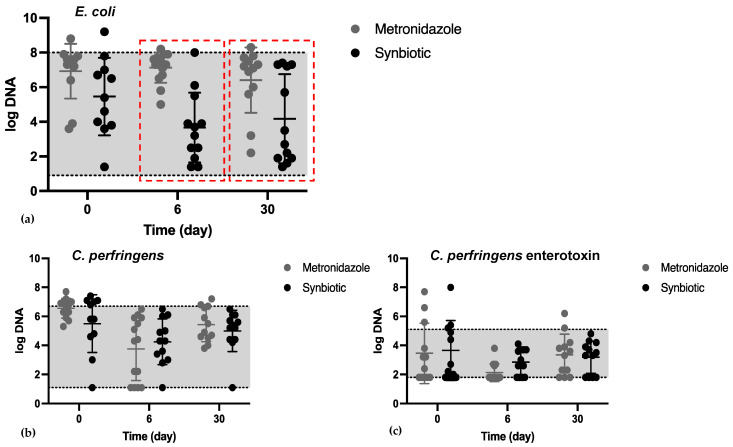
Abundance of *E. coli* (**a**), *C. perfringens* (**b**), and *C. perfringens* strains encoding enterotoxin (**c**) on days 0, 6, and 30. Dots show the mean, error bars show the SD, the *y*-axis represents log DNA/g feces, and the gray area shows the reference interval for the individual bacteria. (**a**) There was a significant difference in the concentration of *E. coli* between the METg and SYNg on day 6 (*p* ≤ 0.0001) and day 30 (red boxes; *p* = 0.01). (**b**,**c**) There was no significant difference in the abundance of *C. perfringens* or *C. perfringens* strains encoding enterotoxin on any of the recorded days.

In 9% of all dogs, the concentration *E. coli* was increased (>8 log DNA/g feces) on day 0. No statistically significant difference in the abundance of *E. coli* was observed on day 0 between the two groups. There was a significant difference between the METg and SYNg regarding the concentration of *E. coli* on day 6 (METg mean 7.1 log DNA/g feces (0.84); SYNg mean 3.6 log DNA/g feces (1.92); *p* ≤ 0.0001) and day 30 (METg mean 6.4 log DNA/g feces (1.78); SYNg mean 4.1 log DNA/g feces (2.46); *p* = 0.01; Figure 4a).

On the day of inclusion, concentration of *C. perfringens* was increased in 35% of all dogs above the reference interval (>6.5 log DNA/g feces). There was no statistically significant difference between the groups in terms of the abundance of *C. perfringens* quantified by qPCR on days 0, 6, or 30. The concentration of *C. perfringens* returned to the reference interval (<6.5 log DNA/g feces) in all dogs in the study on day 6 and in 78% of the dogs on day 30 (Figure 4b) regardless of treatment.

On day 0, in 25% of the dogs in both groups, the abundance of *C. perfringens* strains encoding enterotoxin was increased (>5.1 log DNA/g feces). There was no statistically significant difference in the abundance of *C. perfringens* strains encoding enterotoxin between the two groups on days 0, 6, and 30 (Figure 4c).

On day 0, 3 dogs (13%) were positive for the abundance of *C. perfringens* strains encoding the toxin *NetF*. No dog was positive for *Clostridioides difficile* (*C. difficile*) on day 0.

## 4. Discussion

The results of this prospective, blinded clinical trial showed that, in comparison to a synbiotic, metronidazole had no effect on *C. perfringens* concentration but resulted in a significantly increased concentration of *E. coli*, an increased Dysbiosis Index, and a reduction in *C. hiranonis* concentration. No significant difference could be seen regarding the clinical improvement. Thus, metronidazole had a negative effect on the core microbiome without affecting clinical outcomes.

A recent survey among veterinarians showed that the clinical management of idiopathic acute diarrhea is not consistent with evidence-based recommendations [13,15]. This proportion underscores the continued need for evidence-based recommendations and promotes the dissemination of new information [15]. The present study revealed that for dogs with AD, routine use of metronidazole does not improve the clinical course but has a negative impact on the intestinal microbiome, while synbiotics have similar clinical outcomes but aid in the recovery of the microbiome.

Although the CADS index was lower in the metronidazole group on day 3, there was no significant difference in the diarrhea-specific parameters FC and DF on this day. On all other days, no significant differences were found between the treatment groups. Thus, the results confirmed the findings of previous studies that there is no relevant difference between metronidazole and synbiotic treatment in terms of clinical improvement in dogs with AD [4,7,22].

In addition to the clinical course, the results of the present study showed that AD does not lead to major alterations in the core intestinal microbiota, which was also not observed in other studies evaluating acute diarrhea, including AHDS, in dogs [5,33]. The mild increase in the DI could be related to minor alterations in core microbiota and the increase in *C. perfringens* and *E. coli*. In contrast, major alterations in the core intestinal microbiota and a lack of *C. hiranonis* can be observed in a subset of dogs with chronic enteropathies (CEs) [30,34]. *C. hiranonis* plays a major role in maintaining a normal intestinal microbiome due to its ability to convert primary bile acids to secondary bile acids through 7-α-dehydroxylation [14,19]. A study demonstrated that when the abundance of *C. hiranonis* (in log10) is >4.5, essentially all primary bile acids are converted to secondary bile acids [32]. Secondary bile acids are thought to protect against the growth of several pathogens, including *C. difficile* [32]. Intestinal dysbiosis is linked to CE and often a lower abundance of *C. hiranonis* [30,35]. The presence of *C. difficile* in turn is strongly linked to decreased *C. hiranonis* [35]. In the present study, one dog showed an increased DI with a severely decreased abundance of *C. hiranonis* on the day of presentation, which could suggest dysbiosis associated with subclinical CE [5]. This dog presented 24 months later with chronic gastrointestinal signs and moderate to severe chronic gastritis according to pathohistological findings. In contrast, on day 0, all the other dogs with acute diarrhea had *C. hiranonis* within or slightly below the reference interval, and *C. difficile* was not detected in any of the dogs.

As observed previously, metronidazole administration resulted in a significant decrease in *C. hiranonis* and a significant increase in the DI [19,20,22,36], which was still detectable 3 weeks after the end of treatment in 33% of the dogs. Results indicate that the microbiota tends to return to normal in most dogs; however, the return to pretreatment conditions appears to be individualized. This result supports findings of other studies that also revealed an individual trend toward a return after withdrawal of antibiotic treatment [19,37,38,39]. Therefore, metronidazole has acute and long-lasting effects on the intestinal microbiome in some dogs. Although all dogs in the synbiotic group had a DI < 0 on day 30, 58% of those dogs still showed minor changes on day 30. Thus, while the effect of AD on the intestinal microbiome seems to be mild, minor changes must be assumed at least up to one month after a period of AD.

In contrast to the beneficial bacterium *C. hiranonis*, certain bacterial species, such as *E. coli* and *Clostridium perfringens*, are repeatedly mentioned as potential causes of AD. The results of the present study revealed a self-limiting increase in *C. perfringens* in AD. This finding supports the hypothesis that the increase in *C. perfringens* in AD represents a transient part of mild intestinal dysbiosis [11]. Moreover, no difference in abundance was observed between metronidazole and synbiotic treatment over time; therefore, specific treatment for *C. perfringens* is not needed. Similarly, an increased abundance of *C. perfringens* strains encoding enterotoxin was observed in AD; however, this change also appears to be part of transient intestinal dysbiosis [11]. The pore-forming toxin *NetF* of *C. perfringens* seems to play no major role in dogs with uncomplicated AD, as it was detected in only a few dogs on the day of presentation. Previous studies have shown that *NetF* is more strongly associated with AHDS in dogs, as more than 50% of these dogs harbor these toxigenic *NetF* strains at presentation [33,40,41].

The increase in *E. coli* in the METg can be interpreted as a result of a shift in the bacterial composition due to the effect of metronidazole against anaerobic bacteria, as demonstrated previously [19]. Although *E. coli* is a normal inhabitant of the intestine and most strains are nonpathogenic in most dogs, some strains can be involved in the pathogenesis of gastrointestinal diseases, e.g., histiocytic ulcerative colitis (HUC) in dogs or extragastrointestinal infections in the urinary tract or wounds [42]. The results of the present study showed that in the synbiotic group, the presence of *E. coli* was self-limiting, while metronidazole led to a dysbiosis pattern with increased *E. coli.*

This study has several limitations. First, the owners provided treatments and diet at home. Although the owners were trained in capsule administration, it is possible that the treatments or the diet were not fully administered. Additionally, the owners themselves scored the parameters of the CADS index. Although several variables can be objectively assessed (e.g., defecation frequency and fecal consistency based on the Purina Fecal Scoring Chart), others (e.g., activity) are relatively subjective. The clinical impression also varies based on the time spent with the dog, which differs among owners. A notable limitation of this study is the absence of a placebo group, which prevents comparison with a single supportive treatment. Another limitation lies in the relatively small sample size of dogs within both treatment groups. Nevertheless, the discernible impact of metronidazole on the intestinal microbiome was evident and consistent with prior research. Consequently, it appears improbable that the outcomes would have undergone substantial alteration even with a larger participant pool.

## 5. Conclusions

In conclusion, the results of this prospective, blinded clinical trial comparing metronidazole and a synbiotic in dogs with AD shows that metronidazole causes significant changes on the intestinal microbiome, especially on *C. hiranonis*. This underlines the thoughtful use of metronidazole. Furthermore, the increase in specific bacteria strains, referred to as potential enteropathogens, such as *C. perfringens* and *E. coli*, are self-limiting and normalize simultaneously with clinical improvement and without antibiotic treatment. The synbiotic was not inferior to metronidazole in terms of clinical improvement and should therefore be considered as an antibiotic sparing alternative. These agents can positively influence the self-limiting character of AD via beneficial modulation of the intestinal microbiome and have been safely used for several years However, due to the small number of patients and the lack of a placebo group, further studies are necessary.

## Figures and Tables

**Table 1:** Scoring system for the Canine Acute Diarrhea Severity index (CADS index, maximum 15 points). The severity levels were as follows: nonsignificant (0–3); mild (4–5); moderate (6–8); and severe (≥9).

Parameters	Points			
	0	1	2	3
Activity	normal	mildly decreased	moderately decreased	severely decreased
Appetite	normal	mildly decreased	moderately decreased	severely decreased
Vomiting (times/day)	0	1	2–3	>3
Fecal consistency	normal	moist, shaped	pasty	watery diarrhea
Defecation frequency (times/day)	1	2–3	4–5	>5

**Table 2:** Clinical signs of systemic inflammation, sepsis, or dehydration.

Parameter	Reference Range
Rectal temperature	<37.0 and >39.0 °C [<98.6 °F and >102.2 °F]
Heart rate	>140/min
Hematocrit	>58%
WBC	<5 × 10^9^/L or >20 × 10^9^/L
Banded neutrophils	>1.5 × 10^9^/L

**Table 3:** Sex and breed distribution at baseline in dogs with AD; abbreviations: METg: metronidazole treatment group; SYNg: synbiotic treatment group.

	METg (*n* = 15)	SYNg (*n* = 12)	*p* Value
Sex	7 male, 8 female	4 male, 8 female	0.69
Breeds	Mixed breed (3), Miniature Australian Shepherd (1), Giant Schnauzer (1), Maltese dog (1), Poodle (1), Pug (2), American Bulldog (1), Bichon Frise (1), Border Collie (1), Labrador (1), Yorkshire Terrier (1), French Bulldog (1)	Mixed breed (5), Vizsla (2), Golden Retriever (1), Chihuahua (1), Australian Shepherd (1), Shi Tzu (1), Yorkshire Terrier (1)	0.27

**Table 4:** Bodyweight and age distribution at baseline in dogs with AD; abbreviations: METg: metronidazole treatment group; SYNg: synbiotic treatment group.

	METg (*n* = 15)	SYNg (*n* = 12)	*p* Value
	Median (Range)	Median (Range)	
Bodyweight (kg)	12.7 (5–36.3)	17.2 (5–35.5)	0.31
Age (years)	4.7 (1–13)	5.2 (2–12)	0.81

## Data Availability

The raw data supporting the conclusions of this article will be made available by the authors on request.
